# A possible dual effect of cigarette smoking on the risk of postmenopausal breast cancer

**DOI:** 10.1007/s10654-017-0282-7

**Published:** 2017-07-14

**Authors:** Piet A. van den Brandt

**Affiliations:** 1 Department of Epidemiology, GROW- School for Oncology and Developmental Biology, Maastricht University Medical Centre, PO Box 616, 6200 MD Maastricht, The Netherlands; 2 Department of Epidemiology, CAPHRI- School for Public Health and Primary Care, Maastricht University Medical Centre, Maastricht, The Netherlands

**Keywords:** Breast cancer, Smoking, Menopause, Cohort study

## Abstract

Smoking seems modestly associated with breast cancer, but the potential dual effect of smoking (with opposing properties: carcinogenic vs anti-estrogenic) is understudied. The relationship between smoking before and after menopause and risk of postmenopausal breast cancer was investigated in the Netherlands Cohort Study (NLCS). In the NLCS, 62,573 women aged 55–69 years provided information on smoking, dietary and other lifestyle habits in 1986. Follow-up for cancer incidence until 2007 (20.3 years) consisted of record linkages with the Netherlands Cancer Registry and the Dutch Pathology Registry PALGA. Multivariate case-cohort analyses were based on 2526 incident breast cancer cases and 1816 subcohort members with complete data on smoking. When smoking during pre- and postmenopausal periods was mutually adjusted for, breast cancer risk was significantly positively associated with premenopausal smoking pack-years, but inversely associated with postmenopausal smoking pack-years, both in a dose-dependent manner. In continuous analyses, the hazard ratios (95% CI) were 1.35 (1.10–1.65), and 0.47 (0.28–0.80) per increment of 20 premenopausal, and postmenopausal pack-years, respectively. The interaction between pre- and postmenopausal pack-years in relation to breast cancer risk was significant (*P* < 0.001). This study highlights the importance of distinguishing and adjusting for smoking in different life periods, and suggests dual effects of smoking on postmenopausal breast cancer risk.

**Electronic supplementary material:**

The online version of this article (doi:10.1007/s10654-017-0282-7) contains supplementary material, which is available to authorized users.

## Introduction

The association between smoking and breast cancer remains controversial. A recent meta-analysis [1] found a modest positive association, which was not dependent on including/excluding passive smokers from the reference group. Associations were stronger positive when smoking started early [1], particularly before first birth (e.g. [2, 3]).

However, smoking has carcinogenic properties—through polycyclic hydrocarbons, nitrosamines, and aromatic amines-, and anti-estrogenic properties [4], through inhibiting estrogen production or changing estrogen metabolism [5]. The dual effect of these opposing properties on breast cancer risk is understudied. Only two prospective studies have investigated mutually adjusted effects of smoking pack-years before and after menopause [2, 6]. Due to lower endogenous estrogen production, the anti-estrogenic effect of smoking may become more apparent after menopause and possibly affecting postmenopausal breast cancer risk. This hypothesis was investigated in the Netherlands Cohort Study (NLCS).

## Methods

### Study population and follow-up

The Netherlands Cohort Study (NLCS) started in September 1986 and the female part included 62,573 women aged 55–69 years [7]. At baseline, participants completed a mailed, self-administered 11-page questionnaire on diet, smoking habits, anthropometry, reproductive history, physical activity and other cancer risk factors. The NLCS was approved by institutional review boards from Maastricht University and TNO (Netherlands Organization for Applied Scientific Research). All cohort members consented to participation by completing the questionnaire. Data were processed and analysed using the case-cohort approach, enumerating the cases for the entire cohort, and estimating the person-years at risk in the cohort from a subcohort. This subcohort of 2589 women was randomly sampled from the cohort immediately after baseline and is being followed up for vital status. Follow-up for cancer incidence was established by annual record linkage with the Netherlands Cancer Registry and PALGA, the nationwide Dutch Pathology Registry. After 20.3 years of follow-up, a total of 3354 incident female breast cancer cases were detected. Cases and subcohort members were excluded if they reported a history of cancer (except skin cancer) at baseline and if their smoking data (pack-year level) were incomplete; the selection and exclusion steps are shown in Fig. S1 (supplementary data). There were no relevant differences between included and excluded subjects (data not shown). There were 1816 subcohort members and 2526 breast cancer cases available for multivariable analysis.

### Exposure assessment

Tobacco smoking was addressed through questions on baseline smoking status, and the ages at first exposure and last (if stopped) exposure to smoking, smoking frequency, and smoking duration, for cigarette, cigar, and pipe smokers. Pack-years of smoking were calculated by multiplying the total years of cigarette smoking by the number of cigarettes smoked per day divided by 20. Passive smoking questions related to smoking habits of parents and spouses, exposure to passive smoking at work (past or present), and duration of current daily exposure to passive smoking (open ended question; private and occupational settings combined).

### Statistical analysis

The relationship between smoking and breast cancer risk was evaluated using cox proportional hazards models. Standard errors were estimated using the robust Hubert–White sandwich estimator to account for additional variance introduced by the subcohort sampling [8]. Active cigarette smoking was analyzed as smoking status at baseline (never smokers, ex, current; ever smokers), intensity, duration and pack-years of smoking, age at smoking initiation relative to menarche and first birth, age at cessation and years since cessation for ex-smokers. Trend tests were conducted by fitting ordinal exposure variables as continuous terms, after exclusion of never smokers. Since 98% of never active smokers were exposed to passive smoking by parents, spouse, or at work, it was impossible to exclude passive smokers from the reference category. Estimated pack-years of smoking in different life periods (e.g., before and after menopause) were derived from age at initiation, age at stopping or baseline age, age at menopause, by dividing the total pack-years proportionally over these periods according to duration. In multivariable analyses, hazard ratios (HRs) were corrected for potential confounding by age at baseline (55–59, 60–64, 65–69 years), body height (continuous, cm), BMI (<18.5, 18.5–<25, 25–<30, ≥30 kg/m^2^), non-occupational physical activity (≤30, >30–60, >60–90, >90 min/day), highest level of education (primary school or lower vocational, secondary or medium vocational, and higher vocational or university), family history of breast cancer in mother or sisters (no, yes), history of benign breast disease (no, yes), age at menarche (≤12, 13–14, 15–16, ≥17 years), parity (nulliparous, 1–2, ≥3 children), age at first birth (<25, ≥25 years), oral contraceptive use (never, ever), postmenopausal HRT (never, ever), current smoking status at baseline (no, yes), passive smoking status at work, at home and parental, nutritional supplement use (no, yes), and alcohol intake (0, 0.1–<5, 5–<15, 15–<30, ≥30 g/day).

No adjustment was made for age at menopause, because smoking can induce earlier onset of menopause [6, 9] and the smoking—breast cancer relationship might be mediated by age at menopause. Analyses were repeated after excluding cancers occurring in the first 2 years of follow-up.

Smoking-breast cancer analyses were also conducted within strata of other risk factors; interactions were tested using Wald tests and cross-product terms. In addition, analyses were performed, comparing hormone receptor subtypes of breast cancer. Analyses were conducted using Stata version 12.

## Results

There were 60.7% never, 19.4% ex- and 19.9% current smokers among the subcohort members. Supplementary Table 1S summarizes several baseline characteristics according to smoking status. Ever smokers tended to be younger and leaner than never smokers, while alcohol consumption, education and OC/HRT use was higher. Mean age at menopause was lower in current smokers than never or ex-smokers. Mean age at smoking cessation was 47.1 years; ex-smokers less often reported familial breast cancer, but benign breast disease was more likely. Only 2.4% of never smokers were not exposed to passive smoking by parents, spouse, or at work; this was even lower for ever smokers.

Although statistically significant associations were seen in age-adjusted analyses, baseline smoking status, daily amount, duration and pack-years were not significantly associated with breast cancer risk in multivariable analyses (Table 1), with a HR for the contrast ever versus never smokers, of 1.14 (95% CI 0.98–1.32). After an initial gradual increase in risk with increasing pack-years, the highest exposure category showed a decreased risk, albeit nonsignificant. Age at starting or age at stopping smoking were not significantly associated with breast cancer risk, without a clear trend. An analysis of starting age relative to age at menarche and age at first birth (following Gaudet et al. [10]) showed that the few women who started smoking before menarche were at increased risk (HR = 16.96, 95% CI 4.11–69.89). For those starting after menarche, there was no clear trend in risk with longer periods between starting and first birth.

**Table 1:** Hazard Ratio of breast cancer, according to cigarette smoking characteristics in multivariable-adjusted^a^ analyses, Netherlands Cohort Study 1986–2006

Smoking characteristics	Person-years in subcohort	No. of cases	Age-adjusted		Multivariable-adjusted	
			HR	(95% CI)	HR	(95% CI)
Smoking status						
Never	18,814	1419	1	Ref	1	Ref
Ex-smoker	6547	563	1.16	(1.00–1.36)	1.14	(0.96–1.36)
Current smoker	6080	544	1.22	(1.04–1.43)	1.13	(0.95–1.36)
*P* trend				0.006		0.121
Ever	12,627	1107	1.19	(1.05–1.35)	1.14	(0.98–1.32)
No. of cigarettes/day, ever smokers						
Never	18,814	1419	1	Ref	1	Ref
<5 cigarettes/day	3019	235	1.05	(0.85–1.30)	1.06	(0.82–1.36)
5–<10	2851	243	1.15	(0.92–1.43)	1.11	(0.86–1.43)
10–<15	2214	197	1.22	(0.96–1.55)	1.20	(0.90–1.59)
15–<20	1623	145	1.22	(0.92–1.61)	1.16	(0.84–1.61)
20+	2919	287	1.35	(1.10–1.65)	1.24	(0.95–1.61)
*P* trend (smokers only)				0.084		0.373
Continuous, per 10 cigarettes increment	31,441	2526	1.14	(1.05–1.23)	1.08	(0.98–1.20)
Duration of smoking, ever smokers						
Never	18,814	1419	1	Ref	1	Ref
<10 years	1187	89	1.03	(0.74–1.43)	1.02	(0.71–1.47)
10–<20	2242	195	1.19	(0.93–1.51)	1.22	(0.93–1.60)
20–<30	2848	251	1.21	(0.97–1.50)	1.19	(0.93–1.52)
30–<40	3664	328	1.22	(1.00–1.48)	1.11	(0.86–1.44)
40+ years	2686	244	1.22	(0.98–1.51)	1.04	(0.77–1.42)
*P* trend (smokers only)				0.409		0.672
Continuous, per 10 years increment	31,441	2526	1.05	(1.01–1.10)	1.02	(0.96–1.09)
Pack-years of smoking (total), ever smokers						
0 pack years	18,814	1419	1	Ref	1	Ref
1–9	6168	504	1.11	(0.94–1.30)	1.11	(0.92–1.35)
10–19	2576	233	1.24	(0.99–1.55)	1.18	(0.90–1.56)
20–29	1924	175	1.24	(0.96–1.59)	1.19	(0.88–1.62)
30–39	972	120	1.69	(1.22–2.34)	1.47	(0.99–2.18)
≥40	987	75	1.02	(0.72–1.46)	0.85	(0.56–1.30)
*P* trend (smokers only)				0.235		0.894
Continuous, per 20 pack-years increment	31,441	2526	1.15	(1.04–1.27)	1.05	(0.92–1.21)
Age at initiation of smoking, ever smokers						
Never	18,814	1419	1	Ref	1	Ref
≥26 year	3401	275	1.07	(0.88–1.31)	1.08	(0.84–1.39)
21–25 year	2089	217	1.39	(1.10–1.77)	1.28	(0.96–1.70)
16–20 year	6206	529	1.18	(1.00–1.38)	1.14	(0.93–1.39)
≤15 year	778	78	1.41	(0.96–2.07)	1.16	(0.75–1.81)
*P* trend (smokers only)				0.422		0.969
Initiation relative to first birth in parous, ever smokers						
Never smoker	15,712	1170	1	Ref	1	Ref
Before menarche	33	14	5.95	(1.35–26.18)	16.96	(4.11–69.89)
After menarche, 11+ years before first birth	2049	192	1.30	(1.01–1.67)	1.28	(0.95–1.72)
After menarche, 6–10 years before first birth	3468	274	1.10	(0.89–1.35)	1.02	(0.79–1.31)
After menarche, ≤5 years before first birth	1766	163	1.29	(0.98–1.68)	1.41	(1.02–1.97)
After first birth	3059	196	0.86	(0.69–1.08)	0.91	(0.69–1.20)
*P* trend (smokers only)				0.009		0.116
Time since quitting smoking						
Current	6080	544	1	Ref	1	Ref
Quit 0.1–<5 year	1379	122	0.98	(0.71–1.35)	0.94	(0.65–1.36)
5–<10 year	1185	111	1.04	(0.74–1.46)	1.05	(0.72–1.54)
10–<15 year	1539	97	0.70	(0.51–0.96)	0.66	(0.46–0.96)
15–<20 year	713	73	1.15	(0.76–1.73)	1.13	(0.69–1.85)
Quit 20+ year	1668	156	1.04	(0.78–1.39)	1.15	(0.82–1.61)
*P* trend (ex-smokers only)				0.671		0.209
Age at smoking cessation						
Never smoke	18,814	1419	1	Ref	1	Ref
≤30 year	684	60	1.21	(0.79–1.84)	1.30	(0.82–2.07)
31–40 year	1067	100	1.29	(0.92–1.79)	1.45	(1.01–2.07)
41–50 year	2209	181	1.15	(0.90–1.47)	1.09	(0.82–1.43)
51–60 year	1996	174	1.16	(0.90–1.50)	1.19	(0.90–1.57)
>60 year	528	44	1.04	(0.64–1.67)	0.87	(0.51–1.48)
*P* trend (ex-smokers only)				0.705		0.170

^a^Multivariable analyses were adjusted for: age at baseline (55–59, 60–64, 65–69 years), current smoking status at baseline (no, yes), body height (continuous, cm), BMI (<18.5, 18.5–<25, 25–<30, ≥30 kg/m^2^), non-occupational physical activity (≤30, >30–60, >60–90, >90 min/day), highest level of education (primary school or lower vocational, secondary or medium vocational, and higher vocational or university), family history of breast cancer in mother or sisters (no, yes), history of benign breast disease (no, yes), age at menarche (≤12, 13–14, 15–16, ≥17 years), parity (nulliparous, 1–2, ≥3 children), age at first birth (<25, ≥25 years), oral contraceptive use (never, ever), postmenopausal HRT (never, ever), passive smoking status at work, at home and parental, nutritional supplement use (no, yes), and alcohol intake (0, 0.1–<5, 5–<15, 15–<30, ≥30 g/day)

When smoking during pre- and postmenopausal periods was mutually adjusted, breast cancer risk was significantly positively associated with premenopausal smoking (*P*-trend = 0.003), but inversely with postmenopausal smoking pack-years (*P*-trend = 0.010) (Table 2). In continuous analyses, the HRs (95% CI) were 1.35 (1.10–1.65), and 0.47 (0.28–0.80) per increment of 20 premenopausal, and postmenopausal pack-years, respectively. This inverse relationship with postmenopausal pack-years seemed stronger in never HRT-users and in those with overweight (P-heterogeneity nonsignificant). Further analyses of effect modification by other factors revealed no significant heterogeneity in these associations (data not shown). There was also no significant heterogeneity in these associations, when comparing hormone receptor subtypes of breast cancer, neither was there an effect of exclusion of the first 2 years of follow-up (data not shown).

**Table 2:** Hazard Ratio of breast cancer in relation to pack-years of smoking relevant to menopause in multivariable-adjusted^a^ analyses, Netherlands Cohort Study 1986–2006

Smoking characteristics	Person-years in subcohort	No. of cases	Age-adjusted		Multivariable-adjusted	
			HR	(95% CI)	HR	(95% CI)
Smoking before menopause, pack-years						
0	19,188	1445	1	Ref	1	Ref
1–<5	4281	337	1.07	(0.89–1.29)	1.07	(0.84–1.37)
5–<10	2667	225	1.15	(0.92–1.43)	1.16	(0.87–1.54)
10–<15	1410	135	1.30	(0.98–1.73)	1.29	(0.89–1.88)
15–<20	1434	122	1.17	(0.87–1.56)	1.29	(0.89–1.85)
20+	2298	250	1.51	(1.20–1.89)	1.71	(1.20–2.42)
* P* trend				< 0.001		0.003
Continuous, per 20 pack-years increment	31,278	2514	1.26	(1.10–1.44)	1.35	(1.10–1.65)
Smoking after menopause, pack-years						
0	22,486	1734	1	Ref	1	Ref
1–<5	4904	447	1.20	(1.02–1.42)	0.98	(0.74–1.29)
5–<10	2150	186	1.14	(0.90–1.45)	0.77	(0.53–1.14)
10–<15	1019	95	1.22	(0.87–1.69)	0.75	(0.47–1.19)
15+	719	52	0.92	(0.61–1.38)	0.48	(0.27–0.84)
*P* trend				0.378		0.007
Continuous, per 20 pack-years increment	31,278	2514	1.13	(0.84–1.51)	0.47	(0.28–0.80)
Smoking after menopause without HRT, pack-years						
0	19,795	1507	1	Ref	1	Ref
1–<5	4022	383	1.27	(1.06–1.53)	1.06	(0.77–1.46)
5–<10	1793	158	1.18	(0.91–1.52)	0.80	(0.52–1.23)
10–<15	853	81	1.25	(0.87–1.79)	0.79	(0.48–1.31)
15+	601	42	0.89	(0.56–1.40)	0.47	(0.25–0.89)
*P trend*				0.346		0.008
Continuous, per 20 pack-years increment	27,064	2171	1.12	(0.82–1.55)	0.44	(0.25–0.79)
Smoking after menopause with HRT, pack-years						
0	2691	227	1	Ref	1	Ref
1–<5	882	64	0.88	(0.57–1.36)	0.76	(0.34–1.73)
5–<10	358	28	0.94	(0.51–1.73)	0.96	(0.32–2.86)
10+	283	24	1.02	(0.53–1.98)	0.68	(0.16–2.85)
*P* trend				0.975		0.762
Continuous, per 20 pack-years increment	4214	343	1.13	(0.52–2.43)	1.16	(0.23–5.90)
Smoking after menopause in normal weight, pack-years						
0	11,978	829	1	Ref	1	Ref
1–<5	3061	243	1.16	(0.93–1.45)	1.04	(0.71–1.53)
5–<10	1284	109	1.26	(0.92–1.73)	0.95	(0.56–1.61)
10–<15	526	55	1.51	(0.96–2.35)	1.01	(0.53–1.92)
15+	341	27	1.08	(0.60–1.94)	0.58	(0.26–1.31)
*P* trend				0.076		0.242
Continuous, per 20 pack-years increment	17,190	1263	1.38	(0.92–2.06)	0.58	(0.28–1.17)
Smoking after menopause in overweight, pack-years						
0	10,348	891	1	Ref	1	Ref
1–<5	1776	201	1.34	(1.03–1.75)	0.87	(0.56–1.35)
5–<10	814	74	1.07	(0.73–1.56)	0.56	(0.31–1.04)
10–<15	446	39	1.05	(0.63–1.75)	0.50	(0.24–1.04)
15+	327	25	0.88	(0.48–1.61)	0.40	(0.17–0.93)
*P* trend				0.919		0.012
Continuous, per 20 pack-years increment	13,711	1230	1.06	(0.68–1.65)	0.36	(0.15–0.83)

^a^Multivariable analyses were adjusted for: age at baseline (55–59, 60–64, 65–69 years), current smoking status at baseline (no, yes), body height (continuous, cm), BMI (<18.5, 18.5–<25, 25–<30, ≥30 kg/m^2^), non-occupational physical activity (≤30, >30–60, >60–90, >90 min/day), highest level of education (primary school or lower vocational, secondary or medium vocational, and higher vocational or university), family history of breast cancer in mother or sisters (no, yes), history of benign breast disease (no, yes), age at menarche (≤12, 13–14, 15–16, ≥17 years), parity (nulliparous, 1–2, ≥3 children), age at first birth (<25, ≥25 years), oral contraceptive use (never, ever), postmenopausal HRT (never, ever), passive smoking status at work, at home and parental, nutritional supplement use (no, yes), and alcohol intake (0, 0.1–<5, 5–<15, 15–<30, ≥30 g/day). In the assessment of smoking during a specific period, smoking during the other life periods was adjusted for

**Fig. 1:**
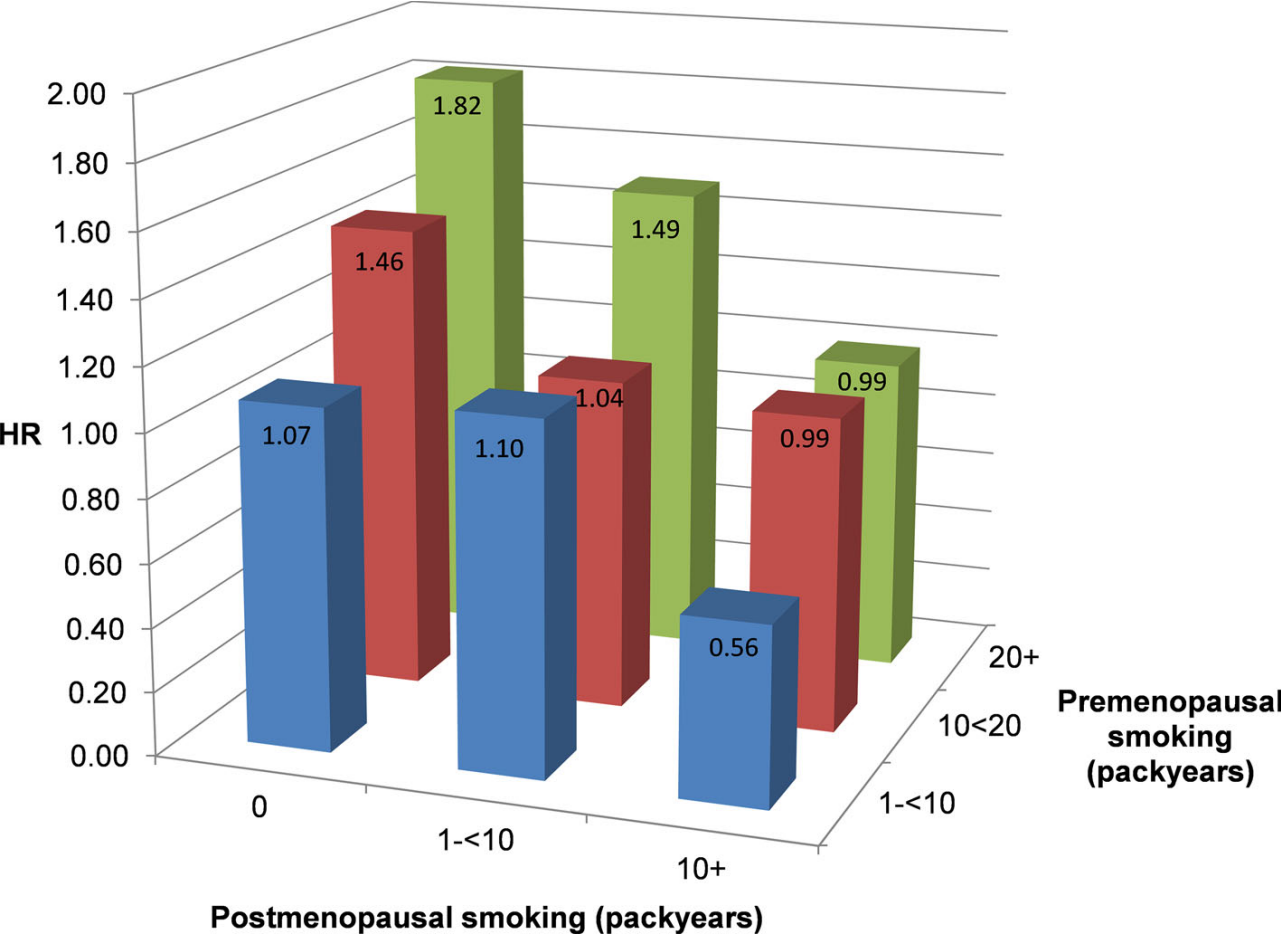
Hazard ratio of breast cancer according to pack-years of premenopausal smoking and pack-years of postmenopausal smoking. *Note* Multivariable analyses were adjusted for: age at baseline (55–59, 60–64, 65–69 years), current smoking status (no, yes), body height (continuous, cm), BMI (<18.5, 18.5–<25, 25–<30, ≥30 kg/m^2^), non-occupational physical activity (≤30, >30–60, >60–90, >90 min/day), highest level of education (primary school or lower vocational, secondary or medium vocational, and higher vocational or university), family history of breast cancer in mother or sisters (no, yes), history of benign breast disease (no, yes), age at menarche (≤12, 13-14, 15–16, ≥17 years), parity (nulliparous, 1–2, ≥3 children), age at first birth (< 25, ≥25 years), oral contraceptive use (never, ever), postmenopausal HRT (never, ever), current smoking status at baseline (no, yes), passive smoking status at work, at home and parental, nutritional supplement use (no, yes), and alcohol intake (0, 0.1–<5, 5–<15, 15–<30, ≥30 g/day)

The interaction between pre- and postmenopausal pack-years in relation to breast cancer risk was highly significant (*P* < 0.001), and is illustrated in Fig. 1, where the HR of breast cancer is presented for various combinations of pre- and postmenopausal pack-years, compared to never smokers. The figure shows that for those who only smoked before menopause, there is an increasing risk with increasing pack-years of smoking: from a HR of 1.07 for 1–10 premenopausal pack-years to 1.82 for 20+ pack-years. However, a decreasing trend in risk is visible with increasing pack-years of postmenopausal smoking. For example, the HR for those with 20+ premenopausal pack-years and 10+ postmenopausal pack-years was 0.99 compared to a HR of 1.82 for women who only smoked before menopause; it was 0.56 for those with 1–10 premenopausal pack-years and 10+ postmenopausal pack-years.

## Discussion

This study showed that pack-years of premenopausal smoking was positively associated, but postmenopausal smoking was inversely related to postmenopausal breast cancer risk, in a dose-dependent manner, when both were taken into account. There was a statistically significant interaction between these factors (antagonism). Baseline smoking status, overall duration and smoking intensity were not significantly related to risk.

A meta-analysis of 27 prospective studies [1] concluded that ever active smoking was modestly, but significantly, associated with breast cancer risk, with no evidence of heterogeneity. The reported SRR of 1.10 is comparable to the HR of 1.14 found here in the NLCS. The meta-analysis reported no differences between subgroups, particularly pre/post-menopause. However, this pre/post-menopause contrast does not refer to the distinction between pre- and postmenopausal smoking, which seems more important [2, 4, 6]. When pack-years smoked in pre- versus postmenopausal periods were mutually adjusted in the NLCS, the opposite associations with breast cancer appeared even stronger than in earlier prospective studies [2, 6], with a significant interaction between pre- and postmenopausal pack-years, suggesting moderately strong opposite effects of smoking: carcinogenic versus anti-estrogenic effects [4]. The Nurses’ Health Study [6] and EPIC [2] were the only two prospective studies that have investigated mutually adjusted effects of smoking pack-years before and after menopause on breast cancer risk.

The anti-estrogenic effect of smoking among postmenopausal women may further reduce their already low circulating estrogen levels [6]. The stronger inverse relationship in women who never used HRT is also compatible with this [2, 6]. In premenopausal years, the anti-estrogenic effect of smoking may not be strong enough to reduce estrogen levels meaningfully, leaving the dominant carcinogenic effect of smoking [4, 6]. The dual effects only appear after mutual adjustment for smoking in different periods, and may explain part of the inconsistencies in the literature on smoking and breast cancer. It might also explain why, in our analysis without mutual adjustment, we observed a decreased breast cancer risk for women exposed to a large number of pack-years (i.e. higher proportion of postmenopausal smoking in the NLCS), while at lower exposure levels a gradual increase in breast cancer risk was seen with increasing pack-years of smoking.

The prospective design and high completeness of follow-up of the NLCS make information bias and selection bias unlikely. In other cohort studies, a further distinction was made between pack-years smoked before and after first birth, and a stronger positive association with the former was found [2, 6]. The NLCS-data did not allow this distinction. However, women who started smoking before first birth, were at increased risk as opposed to those who started after first birth. A further limitation was that there was no update of smoking information after baseline, and that exclusion of passive smokers from never active smokers was impossible, because almost all women had been passively exposed. Nevertheless, we found generally stronger associations than others who did exclude passive smokers (e.g. [2], and the importance of exclusion is not clearly demonstrated in meta-analysis [1].

Anti-estrogenic effects of smoking have been suggested by an earlier age at natural menopause and reduced endometrial cancer risk [5]. Smoking may exert anti-estrogenic effects through nicotinic alkaloids inhibiting aromatase activity and aromatization of androgens into estrogens, the major source of postmenopausal endogenous estrogen production [5], and increased hepatic estrogen clearance [9]. There is evidence that smoking inactivates inactivates estrone, the most important estrogen in the postmenopausal phase. However, there may also be increased formation of genotoxic estrogen metabolites, the (semi)quinones [9]. Given the multiple effects of smoking, further work will be needed to understand the relationships of smoking, estrogen production and metabolism, and carcinogenesis [5].

In conclusion, this study demonstrates the importance of distinguishing and adjusting for smoking in different life periods, and suggests dual effects of smoking on breast cancer, consistent with two other large cohorts [2, 6]. The carcinogenic effect of premenopausal smoking highlights the need for early age prevention programs.

## Electronic supplementary material

Below is the link to the electronic supplementary material.

Supplementary material 1 (DOCX 48 kb)
