# Supplementary material for: A possible dual effect of cigarette smoking on the risk of postmenopausal breast cancer
Source: Eur J Epidemiol. 2017 Jul 14;32(8):683–90. doi: 10.1007/s10654-017-0282-7 (PMC5591344; doi:10.1007/s10654-017-0282-7)
Supplement: Supplementary file 1 — Supplementary material 1 (DOCX 48 kb) [file 10654_2017_282_MOESM1_ESM.docx]

**Supplementary materials**

| Table S1. Baseline characteristics (mean (SD), or percentage) according to smoking status category in subcohort women (in those with complete data on pack-years), Netherlands Cohort Study. | | | |
| --- | --- | --- | --- |
|  |  |  |  |
|  |  | Smoking status |  |
| Characteristic | Never smoker | Ex-smoker | Current smoker |
|  | (n=1431) | (n=458) | (n=469) |
|  |  |  |  |
| Age, mean (yr) | 62.0 (4.3) | 60.9 (4.2) | 60.7 (4.1) |
| Height (cm) | 165.2 (6.2) | 165.2 (6.1) | 165.0 (6.3) |
| BMI (kg/m^2^) | 25.3 (3.5) | 24.9 (3.3) | 24.7 (3.8) |
| BMI age 20 (kg/m^2^) | 21.4 (2.7) | 21.4 (2.7) | 21.4 (2.7) |
| Physical activity (min/day) | 61.1 (53.1) | 72 (53.6) | 62.7 (47.6) |
| Alcohol intake (g/day) | 3.6 (6.9) | 8.6 (10.6) | 9.2 (12.1) |
| Age at menarche (yr) | 13.6 (1.7) | 13.7 (1.8) | 13.7 (1.8) |
| Age at menopause (yr) | 48.8 (4.5) | 48.8 (4.3) | 48.0 (4.9) |
| Number of children (in parous) | 3.6 (2.1) | 3.1 (1.6) | 3.3 (1.9) |
| Age at first birth (in parous, yr) | 27.0 (4.2) | 27.0 (4.2) | 26.0 (4.3) |
| Number of cigarettes per day |  | 10.1 (8.1) | 13.1 (8.1) |
| Duration of smoking (yr) |  | 21.9 (11.4) | 33.7 (10.4) |
| Pack-years of cigarette smoking |  | 11.4 (12.3) | 20.5 (14.6) |
| Age at initiation (yr) |  | 22.9 (8.3) | 24.6 (9.5) |
| Age at cessation (yr) |  | 47.1 (11.1) |  |
|  |  |  |  |
| University or higher vocational education (%) | 7.5 | 13.6 | 9.7 |
| Family history breast cancer (%) | 9.2 | 7.0 | 9.4 |
| History benign breast disease (%) | 7.4 | 9.9 | 7.2 |
| Nulliparous (%) | 17.4 | 17.7 | 19.4 |
| Ever used oral contraceptives (%) | 20.3 | 32.0 | 28.4 |
| Ever used hormone replacement therapy (%) | 10.3 | 18.1 | 13.8 |
| No passive smoking by parents, partner, at work (%) | 2.4 | 1.6 | 0.3 |
|  |  |  |  |
|  |  |  |  |
